# Native Top-Down Mass Spectrometry Combined with High-Resolution Charge Variant Analysis of Trastuzumab Originator and Biosimilars

**DOI:** 10.1021/jasms.5c00438

**Published:** 2026-04-17

**Authors:** Corentin Beaumal, Kristina Srzentić, Sara Carillo, Jonathan Bones

**Affiliations:** † Characterization and Comparability Laboratory, NIBRT − National Institute for Bioprocessing Research and Training, Foster Avenue, Mount Merrion, Blackrock, Dublin A94 X099, Ireland; ‡ Thermo Fisher Scientific, 11 Neuhofstrasse, 4153 Reinach, Switzerland; § School of Chemical and Bioprocess Engineering, University College Dublin, Belfield, Dublin D04 V1W8, Ireland

**Keywords:** native top-down MS, charge variant analysis (CVA), intact monoclonal antibodies (mAbs), proton-transfer charge reduction (PTCR)

## Abstract

Comprehensive characterization of monoclonal antibody (mAb) charge heterogeneity is essential to ensure product quality, maintain batch consistency and support biosimilar development. Charge variant analysis (CVA) is widely used to separate acidic and basic proteoforms from the main species. However, cation-exchange chromatography coupled to mass spectrometry provides limited information and cannot localize the post-translational modifications (PTMs) responsible for mAb heterogeneity. Here, the coupling of pH-gradient CVA with native top-down mass spectrometry (TD-MS) for proteoform-specific analysis of trastuzumab is presented. Individual charge variants were chromatographically separated under native conditions and directly fragmented on the chromatographic time scale using higher-energy collision dissociation (HCD), electron-transfer dissociation (ETD) and ultraviolet photodissociation (UVPD). The addition of proton-transfer charge reduction (PTCR) helped reduce spectral congestion and enhanced the detection of high-mass fragment ions, resulting in improved sequence coverage. This workflow enabled the complete sequencing of the complementarity-determining region (CDR) 3 and the direct identification and insights into the location of key PTMs at the intact-protein level, including deamidation, succinimide and N-terminal pyroGlu for individual proteoforms. Comparison of five trastuzumab samples (originator and biosimilars) demonstrated high reproducibility in fragmentation patterns, sequence coverage and variant assignment, highlighting the robustness of the method. Although limitations remain due to the challenges of fragmenting intact mAbs under native conditions, this work establishes a proof of concept for CVA native TD-MS characterization of mAbs to complement bottom-up and middle-down analyses, and has potential for broad applicability for antibody-based biopharmaceuticals.

## Graphical abstract

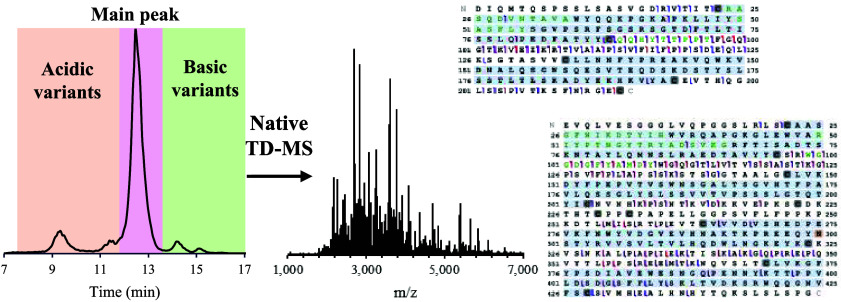

## 1 Introduction

Monoclonal antibodies (mAbs) are one of the most complex and successful classes of biopharmaceuticals, with more than 130 mAbs approved by the Food and Drug Administration (FDA) and/or the European Medicines Agency (EMA) as of 2025.^1,2^ These therapies are used to treat a wide range of diseases, including cancer and autoimmune disorders.^3–5^ The structural complexity of mAbs arises from the various types of post-translational modifications (PTMs) that they can undergo during their production and purification, leading to a heterogeneous mixture of proteoforms.^6,7^ PTMs, such as glycosylation, deamidation and C-terminal lysine clipping can impact mAb stability, biological activity, pharmacokinetics and immunogenicity, and are considered critical quality attributes (CQAs) by the regulatory agencies.^8–10^ Thus, comprehensive analytical characterization of mAb charge heterogeneity is essential to ensure product quality and batch-to-batch consistency.^11^ In addition, the end of patent protection for some biotherapeutics has paved the way for the development of biosimilars, which mostly rely on the clinical data from the originator molecule for regulatory approval. Consequently, employing detailed and precise characterization methods is crucial to confirm their biosimilarity.^12–14^

Charge variant analysis (CVA), typically performed by imaged capillary isoelectric focusing (icIEF) or cation exchange chromatography (CEX), is the standard method for assessing charge heterogeneity in mAbs.^15–18^ In pH gradient-based CEX methods, species separation is based on their isoelectric point (pI), with elution occurring when the pH of the mobile phase reaches the pI of the proteoform, allowing separation of acidic and basic variants from the main peak.^19,20^ Common acidic species typically originate from the deamidation of asparagine residues and the presence of sialic acids.^21^ Other modifications, such as glycation or nonclassical disulfide linkages, have also been observed to generate acidic species. On the other hand, well-known modifications, including the presence of unprocessed C-terminal lysine,^22,23^ C-terminal amidation,^24^ N-terminal glutamine^25^ and succinimide intermediate formation,^26^ result in basic species. Despite their effectiveness, CEX methods alone provide limited structural insight and cannot localize or directly confirm the modifications responsible for charge heterogeneity. Recent advances in volatile buffers and ion transmission within the mass spectrometer, along with higher sensitivity and specificity, have increased interest in native mass spectrometry (nMS) for the analysis of intact mAbs. Hyphenation of CVA to nMS enables confident elucidation of mAb charge variant identity and facilitates the evaluation of PTMs.^27–29^ However, CVA followed by intact mass nMS provides only insights into the PTMs present on the molecule, but information about their location in the primary sequence is not available.

A widely used approach for site-specific identification of PTMs is bottom-up peptide mapping, which involves enzymatic digestion before LC-MS analysis. While peptide mapping is a powerful approach for identifying PTMs, protein structural integrity is disrupted, resulting in a loss of information about the coexistence of PTMs at the intact level within individual proteoforms.^30^ In contrast to peptide mapping, top-down (TD-MS) and middle-down (MD-MS) mass spectrometry, in which intact mAbs or their subunits, respectively, are directly fragmented in the gas phase, have emerged as promising approaches for the comprehensive analysis of mAb heterogeneity at the proteoform level.^30^ Multiple studies (recently reviewed by Khristenko et al.^31^) have been published demonstrating the capabilities of MD-MS to obtain significant sequence coverage of mAb subunits,^32^ which facilitates the identification of PTMs^33,34^ or the confirmation of drug location in ADCs^35–37^ for example. Notably, this has been possible thanks to the use and combination of multiple fragmentation techniques,^38–42^ such as higher-energy collision dissociation (HCD), electron-transfer dissociation (ETD), electron-capture dissociation (ECD) and ultraviolet photodissociation (UVPD), as well as the use of proton-transfer charge reduction (PTCR) to reduce spectral complexity and improve the sequence coverage.^37,38,40^ Fewer studies report the characterization of intact mAb by TD-MS, i.e., without digestion into subunits.^43–46^ Indeed, the fragmentation of high-molecular weight molecules, such as intact mAbs (∼150 kDa), represents a significant challenge, especially under native conditions, arising from signal dispersion from the precursor ion into many more channels, including multiple charge states of a single fragment. Recently, several studies using native TD-MS, i.e., where intact proteins were fragmented under native conditions, for various purposes have been published,^47–50^ demonstrating the growing interest in this approach. However, only a few of them have reported the characterization of mAbs,^51–54^ and all have used direct infusion, which allows for long acquisition times but can prevent the identification of low-abundant proteoforms.

Trastuzumab, a humanized IgG1 monoclonal antibody targeting the HER2 receptor, was used as a model for this study.^55,56^ Here, we report the application of native TD-MS coupled with pH gradient-based charge variant analysis for the high-resolution characterization of the trastuzumab originator drug product material and direct comparison with market-authorized biosimilars. Using this approach, individual charge variants were chromatographically separated and analyzed under native conditions, enabling direct association of each CVA-resolved species with its intact proteoform and allowing mass determination, along with direct proteoform-specific fragmentation on the LC time scale. We demonstrate the identification and insights about the location of PTMs, including deamidation, succinimide, and N-terminal pyroglutamate formation. We also assess the reproducibility of fragmentation and the benefits of using complementary fragmentation techniques (HCD, ETD, and UVPD) to provide information about key regions of mAbs, such as the complementary-determining regions (CDRs). Furthermore, the implementation of proton transfer charge reduction (PTCR) improved fragment ion detection and sequence coverage, providing deeper insights into the charge heterogeneity. This work establishes a proof-of-concept for coupling CVA and native TD-MS to achieve comprehensive, high-resolution charge-variant analysis of therapeutic antibodies and their biosimilars.

## 2 Experimental Section

### 2.1 Chemicals and Reagents

Ammonium bicarbonate (LC-MS grade), and ultrapure water (Optima, LC-MS grade) were purchased from Fisher Scientific (Dublin, Ireland). Ammonium hydroxide 1 M solution and acetic acid (99%, trace metal basis) were obtained from Sigma-Aldrich (Wicklow, Ireland). The monoclonal antibody trastuzumab and all biosimilars (21 mg/mL) were commercially sourced from Evidentic (Berlin, Germany). The ProPac 3R SCX (2 × 50 mm, 3 μm particle size) HPLC column was obtained from Thermo Scientific (Sunnyvale, CA, USA).

### 2.2 Strong Cation Exchange (SCX) Chromatography–Mass Spectrometry Analysis

SCX-UV-MS experiments were performed on a Thermo Scientific Vanquish Horizon instrument equipped with a column compartment, a binary pump H and a split sampler FT (Thermo Scientific, Germering, Germany). Mobile phases were 25 mM ammonium bicarbonate and 30 mM acetic acid in MS-grade water as A, and 10 mM ammonium hydroxide in MS-grade water as B. The pH of A and B were 5.3 and 10.9, respectively, no adjustments were made. Charge variant separation was performed using a ProPac 3R SCX (2 × 50 mm) HPLC column (Thermo Scientific, Sunnyvale, CA, USA), and a 40–95% B gradient was applied in 15 min. The flow rate was 0.2 mL/min for all experiments; the column oven temperature was held at 25 °C, and UV detection was performed at 280 nm. For MS detection, the Vanquish Horizon was hyphenated to the Ion Max NG ion source of an Orbitrap Ascend BioPharma Edition Tribrid MS instrument (Thermo Scientific, San Jose, CA, USA) with the Native MS option allowing mass detection up to *m*/*z* 16,000. For all LC-MS experiments, 30 μg (1.5 μL) of trastuzumab was injected directly from the formulation buffer. MS analysis was performed selecting the intact protein mode, using high-pressure (20 mTorr) and high mass range settings. The spray voltage was 3.8 kV, sheath and aux gas settings were 25 and 10 arbitrary units, respectively, the vaporizer temperature was set to 200 °C, and the ion transfer tube was set to 275 °C, the RF-lens level was set to 100%. Acquisition was performed in positive mode, with an in-source collision-induced dissociation (CID) of 120 V, 10 microscans, a resolving power of 30,000 (at *m*/*z* 200), an AGC target of 2 × 10^5^, a maximum injection time of 59 ms, and a scan range of *m*/*z* 2000–10,000.

### 2.3 Strong Cation Exchange Chromatography Top-Down Mass Spectrometry Analysis

SCX top-down MS experiments were performed using the same separation conditions as described in the previous section, except that the UV detector was bypassed. Targeted LC-MSn experiments were performed on an Orbitrap Ascend BioPharma Edition Tribrid mass spectrometer equipped with UVPD, ETD, and PTCR options in intact protein mode using the high-pressure (20 mTorr) setting. All spectra were recorded in “full profile” mode at a resolving power of 240,000 (at *m*/*z* 200) and by averaging 5 microscans per spectrum. MS source parameters were the same as for the SCX-MS analysis described above. A single precursor charge state (27+) was selected using a 100 *m*/*z* quadrupole isolation window (facilitating selection of all the glycoforms present in a single charge state) for fragmentation by HCD, ETD, and UVPD. After fragmentation, 3000 *m*/*z* wide-window isolation of the fragment ion population, centered on 2800 *m*/*z* (center of the fragment ion population), was performed in the ion routing multipole (HCD value set to 0% NCE) to improve signal quality. For ETD MS2 experiments, the fluoranthene reagent anion target was set to 1 × 10^6^, the maximum ETD reagent injection time to 200 ms, and reaction times between the ETD reagent and precursor ions of 25 and 45 ms were used. For HCD MS2, normalized collision energies (NCE) of 40 and 45% were used. UVPD MS2 experiments were performed using a solid-state 213 nm Nd: YAG laser with irradiation times of 30 and 35 ms. PTCR MS3 was performed after ETD 25 ms and UVPD 30 ms experiments only, using a 3000 Th isolation window (1300–4300 Th). PTCR reagent (perfluoroperhydrophenantrene) target was set to 1 × 10^6^ and a 20 ms reaction time between PTCR reagent and precursor ions was used. For all experiments, AGC target was set to 5 × 10^5^, maximum injection time to 500 ms and the acquisition mass range was set to *m*/*z* 500–8000 for MS2 experiments and *m*/*z* 1000–10,000 for PTCR MS3 experiments.

### 2.4 Tryptic Digest LC-MS/MS Analysis

To provide PTMs levels through an orthogonal assay, peptide mapping was performed on all samples. Briefly, 100 μL for each sample were digested with trypsin and analyzed by LC-MS/MS using a Vanquish Flex HPLC equipped with a Hypersil Gold Peptide column (Thermo Scientific, Sunnyvale, CA, USA) and hyphenated to a Thermo Scientific Orbitrap Exploris 240 mass spectrometer (Thermo Scientific, Bremen, Germany). Full details for the sample preparation, sample analysis and data processing were performed as described previously.^57^ Triplicate analyses were performed for each sample.

### 2.5 Data Analysis

Peak integration was performed using Chromeleon 7.3.2 (Thermo Scientific, Germering, Germany) software using the quantitative MS processing method. SCX-MS raw files were exported to BioPharma Finder (BPF) 5.3 software (Thermo Scientific, San Jose, CA, USA) and processed using the ReSpect deconvolution algorithm (for isotopically unresolved data). Detailed parameters are described in Table S1 (Supporting Information). Top-down raw files were analyzed using TDValidator software (Proteinaceous, Evanston, IL, USA), relying on an isotopic distribution fitting algorithm to match theoretical fragment ions derived from the chemical formula of the proteoform-of-interest.^58^ The following parameters were used for data processing: the signal-to-noise ratio (S/N) threshold was set to 10, fragment tolerance to 10 ppm, subppm tolerance to 3 ppm, cluster tolerance to 0.35. The charge state range was 1–25+, and minimum score threshold was set to 0.60. For HCD fragmentation, *b*/*y* ions were searched, while *c*/*z* ions were selected for ETD and *a*/*a+*/*x+*/*y-* for UVPD. Identified fragment ions were further manually validated. Details on the time window selected for each proteoform can be found in Table S2 (Supporting Information).

## 3 Results and Discussion

### 3.1 Intact Mass Analysis

Charge variant analysis (CVA) is a powerful technique for deciphering mAb heterogeneity. Here, a pH gradient was utilized, with elution occurring when the gradient pH equals the pI of any particular charge variant present.^15^ Under traditional convention, when performing cation exchange chromatography (CEX), species eluting prior to the main peak are referred to as acidic variants and those eluting after the main peak as basic variants.^28^

Trastuzumab originator and biosimilars were characterized using a pH gradient-based CEX as described in Füssl et al.^16^ Base peak chromatograms obtained from trastuzumab originator and biosimilars are displayed in Figure 1A, showing good chromatographic resolution. Similar elution profiles were observed, with the presence of the main variant eluting at 12.5 min, and the presence of lower abundance acidic and basic species eluting before and after the main peak, respectively. The main peak, identified as intact trastuzumab with processed C-terminal lysine residues on both heavy chains (Table S3, Supporting Information), accounts for 78–94% of the total identified species (Figure 1B). Two major acidic species can be observed, both having a +1 Da mass shift relative to trastuzumab with processed C-terminal lysine residues on both heavy chains. The most abundant one, eluting around 9.3 min, was identified as trastuzumab bearing one deamidation (+0.98 Da) and represents between 5.6 and 8% of all the identified variants, except for Ogivri, where a lower level of deamidated trastuzumab (1.9%) was found (Figure 1B). In addition, another low-abundant variant with a +1 Da mass shift eluted just before the main peak, at approximately 11.5 min. Based on the mass shift and retention time, this variant can be identified as deamidated trastuzumab with isomerization of the aspartic acid residue (Asp). Indeed, while isomerization of Asp does not affect the mass of the molecule, it has been widely described in the literature to impact the local tertiary structure of the antibody, changing its surface characteristics and resulting in an increased retention time.^59,60^ The total level of deamidation was confirmed by peptide mapping, performed in triplicate for all samples (Figure S1, Supporting Information).

**Figure 1:**
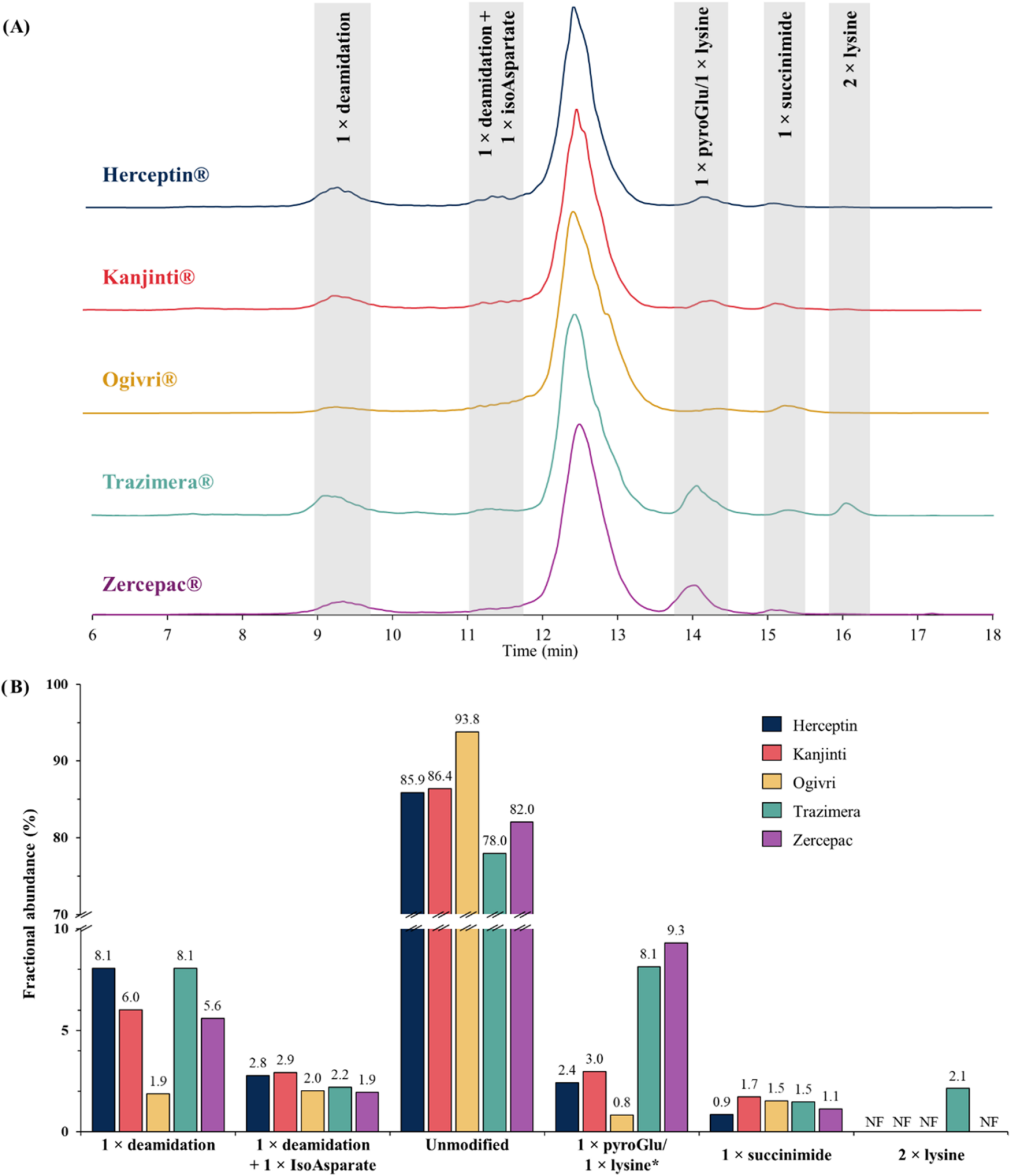
(A) Base peak chromatograms of trastuzumab originator and biosimilars. The main acidic and basic charge variant species identified are highlighted in gray. (B) Fractional abundance of each identified species for all the samples, based on the integration of the base peak chromatograms.

Species identified in the basic region of the chromatogram (i.e., eluting after the main peak) include trastuzumab with pyroGlu (−17 Da, 14.1 min), succinimide (−18 Da, 15.3 min) modifications, as well as one unprocessed lysine (+128 Da, 14.3 min) and two unprocessed C-terminal lysine residues (+256 Da, 15.9 min) variants. Levels of succinimide (0.9–1.7%) and pyroGlu (0.8–3%) species (from base peak chromatogram integration, MS arbitrary units) were comparable between the samples (Figure 1B) and in line with peptide mapping results (Figure S1, Supporting Information). On the contrary, lysine variants (i.e., trastuzumab with one or two unprocessed C-terminal lysine) were only observed for two of the biosimilars, Trazimera and Zercepac. While trastuzumab with an unprocessed C-terminal lysine was found in both biosimilars, the two unprocessed lysine variant was detected only in Trazimera (2.1%, Figure 1B). Those results demonstrate the similarity of the trastuzumab biosimilars to the originator molecule, as mainly the same variants were found in comparable proportions. The additional lysine variants identified in some biosimilars, while not found in the originator, are known variants in trastuzumab and have been extensively studied in the literature.^61–63^ It has been demonstrated that lysine variants in IgG1 do not affect its stability, potency, or its structure and have been ranked as “low” or “very low” risk CQAs.^62,64,65^

In addition to the comparison of the charge variants, differences in the N-glycan distribution on trastuzumab samples were also studied. The glycosylation profile of the released antibody is key information and usually a CQA that is monitored to demonstrate consistency between batches. Indeed, a study by Luo et al. showed that among more than 150 recombinant antibodies approved by the FDA, 45% had glycosylation specifications for the release testing of the drug substance.^66^ Deconvolution of the main peak was performed using BPF 5.3 software, allowing for the determination of the relative intensity of each glycoform (Figure 2). Briefly, the G0F_G1F glycoform was the most abundant in all the samples except for Zercepac, where the G0F_G0F glycoform was prominent. In all samples, glycoforms G0_G0F, G0F_G0F, G0F_G1F, G1F_G1F, G1F_G2F, and G2F_G2F were detected in varying proportions, as detailed in Table 1. Complex fucosylated glycans are common glycan species found in mAbs and essential for their function.^66^ The combined abundance of the fucosylated glycans was also in line with the abundances observed in other FDA-approved IgG1 samples. Additionally, the G0_G0 glycoform was identified in all samples except Ogivri, where the M5_G0F glycoform was found. Finally, the low-abundant high-mannose glycoform M5_M5 was also found in Herceptin, Kanjinti and Zercepac, with relative intensities of 0.9, 1.3, and 5.7%, respectively (Table 1). This glycoform is not intentionally produced as it is associated with increased clearance and reduced half-life.^67^ While upstream and downstream processes are optimized by manufacturers to minimize their formation, small amounts reflecting unavoidable byproducts of mAb production can be detected in drug products, which are acceptable as long as they remain within established regulatory guidelines and do not affect clinical performance.^66^ Glycation was checked by peptide mapping (Figure S2A, Supporting Information). As only minor differences in glycation levels were observed, all the differences observed can be mainly attributed to N-glycan populations (Figure S2B, Supporting Information).

**Figure 2:**
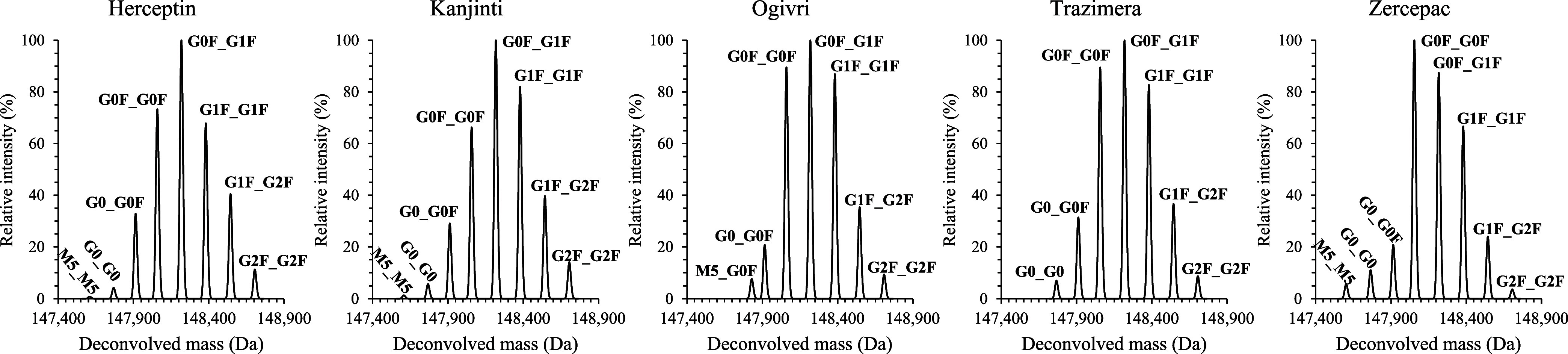
Deconvolved masses of the main elution peak of trastuzumab for each sample and the associated glycoforms identified.

**Table 1:** Trastuzumab Glycoforms Identified in the Main Elution Peak for Each Sample

		Herceptin		Kanjinti		Ogivri		Trazimera		Zercepac	
Trastuzumab glycoform	Theoretical mass (Da)	Relative intensity (%)	Fractional abundance	Relative intensity (%)	Fractional abundance	Relative intensity (%)	Fractional abundance	Relative intensity (%)	Fractional abundance	Relative intensity (%)	Fractional abundance
M5_M5	147,600.07	0.9	0.3	1.3	0.4	NF	NF	NF	NF	5.7	1.8
M5_G0F	147,828.32	NF	NF	NF	NF	7.7	2.2	NF	NF	NF	NF
G0_G0	147,764.29	4.3	1.3	5.7	1.7	NF	NF	7.0	2.0	11.1	3.5
G0_G0F	147,910.43	32.9	9.9	29.2	8.6	20.8	6.0	31.5	8.8	20.9	6.5
G0F_G0F	148,056.57	73.3	22.1	66.3	19.6	89.6	25.6	89.5	25.1	100.0	31.3
G0F_G1F	148,218.71	100.0	30.2	100.0	29.5	100.0	28.6	100.0	28.1	87.5	27.4
G1F_G1F	148,380.85	67.9	20.5	82.0	24.2	86.9	24.8	82.7	23.2	66.1	20.7
G1F_G2F	148,542.99	40.6	12.2	39.8	11.8	35.5	10.1	36.7	10.3	24.1	7.5
G2F_G2F	148,705.13	11.4	3.4	14.2	4.2	9.4	2.7	8.8	2.5	3.7	1.1

### 3.2 Native Top-Down Analysis of the Main Species

Charge variant analysis enabled the separation of different proteoforms, which were subsequently identified by deconvolving the MS signal from each chromatographic peak. However, this approach is unable to provide information on the localization of modifications present in the sequence. Peptide-centric approaches (*i.e*., peptide mapping) are usually required to generate such information. However, such methods are time-consuming and information related to the intact protein structure, such as the coexistence of PTMs, is lost.^30^ As an alternative strategy, performing top-down MS following CVA would allow fragmentation of proteoforms on the LC time scale and provide information on the location of modifications. As a proof-of-concept, we developed a native TD-MS method for characterizing trastuzumab proteoforms on the LC time scale. For all samples, the most abundant trastuzumab charge state (27+) was isolated using a 100 Th isolation window, encompassing all the glycoforms of the selected charge state in the quadrupole, utilizing its high *m*/*z* isolation capabilities. Then, precursors were submitted to HCD, ETD, and UVPD fragmentation. Examples of the fragmentation spectra obtained for each fragmentation technique are displayed in Figure S3 (Supporting Information). MS/MS were acquired in full profile mode and averaged over each chromatographic peak. Averaging full profile mode data improves the S/N ratio, resulting in higher spectral quality data and more confident fragment ion annotations. Sequence coverage obtained for each fragmentation technique is shown in Figure 3. Bar plots describe the average sequence coverage of the light and heavy chain for the 5 samples using each fragmentation technique. Detailed results for each sample (i.e., originator and each biosimilar) are available in Figure S4 (Supporting Information). On average, ETD-based fragmentation provides sequence coverage of 7–13%, 9–13% using HCD and 4–12% using UVPD for the heavy chain and the light chain, respectively. When combining all the fragmentation techniques, average sequence coverage of 30% is observed for the light chain and 21% for the heavy chain. In addition, we noticed that the identified fragments were mainly located outside the intrachain disulfide bridges, with only a few of them being identified within the disulfide bridges (Figure 3C,D). Importantly, native TD allowed for complete sequencing of the CDR3 on both chains, a key region involved in antigen binding.^68^

**Figure 3:**
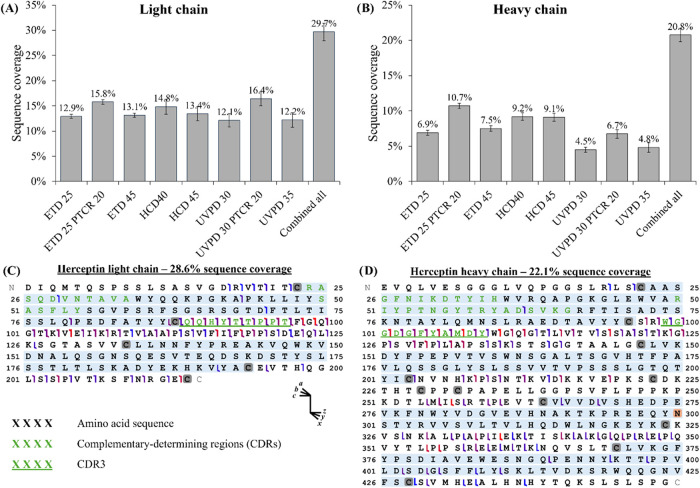
Bar plot representing the average sequence coverage obtained for the (A) light chain and (B) heavy chain without lysine, by each fragmentation used in this study as well as the sequence coverage after combination of all the fragmentation together (*n* = 8). The average is based on the sequence coverage obtained for each sample (*n* = 5) and error bars represent the standard deviation. Example of fragmentation map of the (C) light chain and (D) heavy chain of trastuzumab originator. Complementarity-determining regions (CDRs) are displayed in green letters, and Fc glycosylation site highlighted in orange. Cysteines involved in disulfide bridges are highlighted in gray and the amino acids within the intrachain disulfide bridges in light blue.

Although sequence coverage appears limited, several parameters should be considered. First, precursor ions selected for fragmentation are high *m*/*z* ions of an intact mAb, typically around 150 kDa. Consequently, well-known limitations related to the size of the molecule and difficulties in fragmenting high-molecular-mass proteins apply.^69,70^ Second, experiments were performed under native conditions, introducing additional technical considerations and challenges of conducting top-down MS experiments, relative to denaturing conditions. Due to the use of native conditions, disulfide bridges, which are crucial for maintaining mAb integrity, were not reduced. Accordingly, fragmentation of the sequence by ETD or HCD within intrachain disulfide bridges (and identification of the potential fragments generated) is unlikely.^43,71,72^ This is in line with our observation for both chains, having identified fragments mostly found outside of the disulfide bridges (Figures 3B,C, and 4). The maximum sequence coverage that can be obtained considering only amino acids outside of disulfide bridges is 41 and 42%, for the light and heavy chains, respectively. Consequently, the sequence coverage that we obtained represents 74% of the maximum sequence coverage attainable for the light chain and 50% for the heavy chain. Another challenge of working under native conditions is the limited number of charges available. Here, the charge states range from 25+ to 30+, with 27+ being the most abundant. In contrast, for an intact mAb under denaturing conditions, charge states range from 40+ to 70+, with a maximum abundance around charge state 55+. The limited number of charges under native conditions reduces fragmentation efficiency, especially for ETD, where reaction efficiency is strongly correlated with the precursor charge state,^73,74^ and for HCD, where the low number of protons results in less evenly distributed fragmentation compared to denaturing conditions.^75,76^ Native-like conditions also preserve the folded conformation of the mAb. Consequently, while under denaturing conditions the protein is widely unfolded, with most of the sequence exposed, in its native state, the mAb folded structure results in some parts of the sequence being buried and less accessible for fragmentation.^77–80^ In addition, native-like conditions yield lower charge state precursors than denaturing conditions. High charge states from denaturing conditions often produce broader sequence coverage across multiple cleavage sites, as enough mobile protons are present to induce relatively unspecific fragmentation.^81^ On the contrary, native-like conditions generate lower chare states, which yield to more localized fragmentation on the sequence (e.g., N-term of proline or C-term of aspartic acid residues).^76,82^ This behavior, especially observed for collision-induced fragmentation, is related to the limited mobility of the proton in the case of low charge states. Finally, those experiments are performed on the LC time scale, meaning the number of scans that can be acquired is limited by the elution peak width. Indeed, one of the key parameters in TD-MS is the S/N ratio, as a high S/N ratio allows the identification of more fragment ions.^83,84^ For example, the main elution peak allows for the acquisition of approximately 25 scans when performing HCD, 20 scans using ETD and UVPD and only 15 scans when PTCR is used, thereby limiting the signal-to-noise (S/N) ratio of the fragment ions.

**Figure 4:**
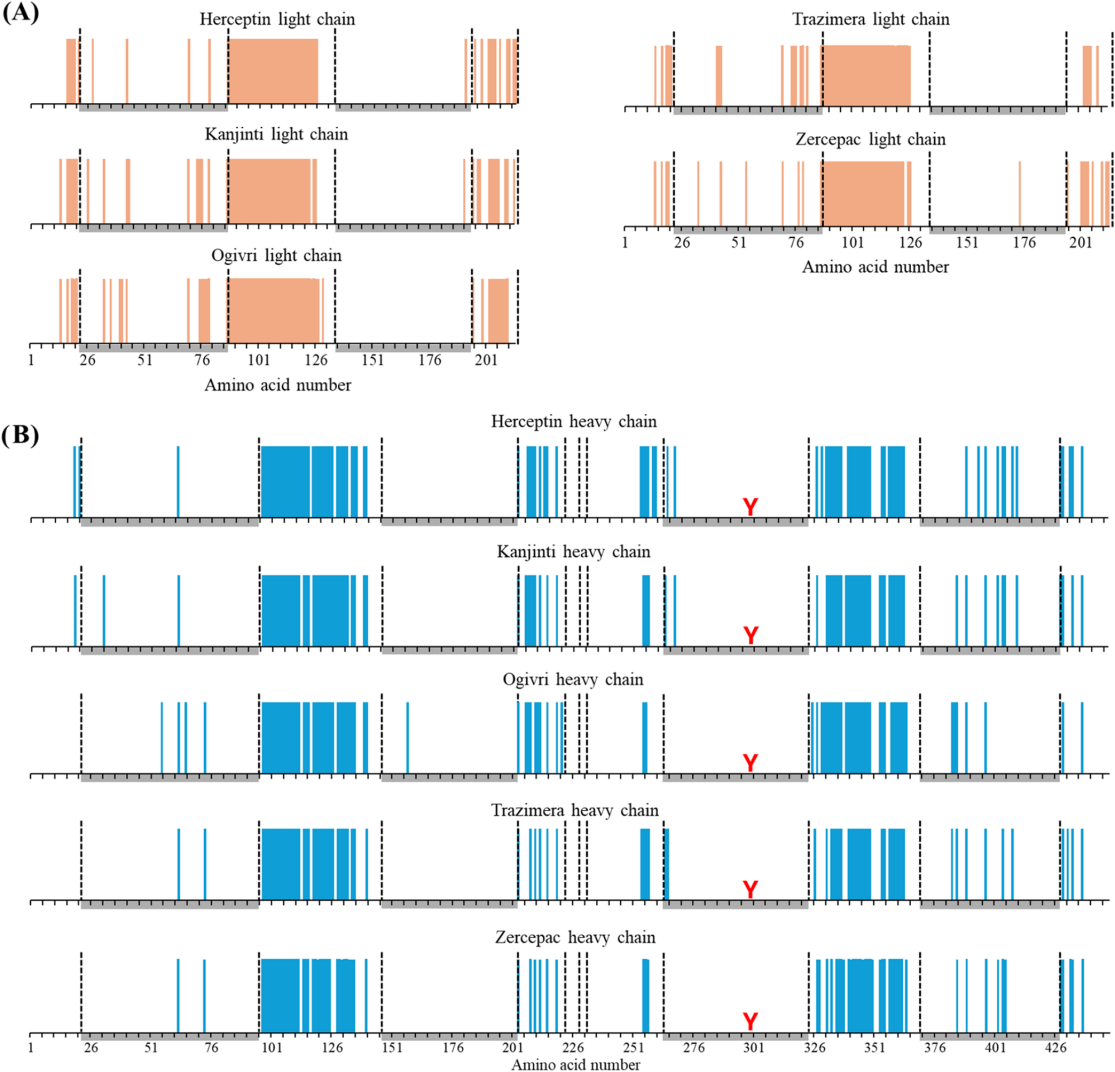
Bar code fragmentation maps of trastuzumab (A) light chains in orange and (B) heavy chains in blue of each sample. Cysteines in the sequence are represented by the vertical black lines and the region of the sequence underlined in gray depict the inside of the intrachain disulfide bridges. Glycosylation site of Asn 300 in heavy chain is represented by the red Y-shape.

In addition to these observations, the comparison of the identified fragments and the sequence coverage demonstrate a good reproducibility between the samples. Standard deviation between the five samples on the sequence coverage is <1 and <1.5% for the heavy and light chains, respectively (Figure 3A,B). Similarly, the locations of the identified fragments are comparable between the samples, as depicted in the bar code fragmentation maps in Figure 4. These results highlight the ability of the native TD approach to provide reproducible sequence information across different samples. It can be noted that EThcD was not included in this study but remains an appropriate option to consider for improving sequence coverage.

### 3.3 Improved Sequence Coverage in Native TD When Using PTCR

As demonstrated for the main peak, conventional fragmentation techniques yield high-quality fragmentation spectra, enabling the identification of fragment ions on an intact mAb under native conditions. However, among the fragmentation techniques used, ETD and UVPD are known to lead to highly informative spectra that are often congested, preventing the identification of all fragment ions generated.^37,40,85,86^ To circumvent this issue and identify as many fragments as possible, PTCR can be applied to reduce the charge states of fragment ions via ion–ion reactions. During the PTCR reaction, the most highly charged species undergo charge reduction first, leading to an increase in their *m*/*z* values (i.e., lower charge state). This allows to effectively redistribute ions across the *m*/*z* range, reducing spectral congestion and improving fragment detection. We employed this strategy after ETD and UVPD fragmentation, which generated the most congested spectra with many overlapping peaks, by applying a 20 ms PTCR reaction time. On average, the use of PTCR after ETD led to an increase in sequence coverage by 22% for the light chain (15.8% vs 12.9%) and 49% for the heavy chain (6.7% vs 4.5%), and by 35 and 55% after UVPD for the light (16.4% vs 12.1%) and heavy (10.7% vs 6.9%) chains, respectively (Figure 3A,B).

In addition to improvements in sequence coverage, PTCR enables the detection of fragment ions present in the fragmentation spectrum but not identified due to their low abundance and split signal across multiple channels, as well as spectral congestion. By redistributing the charges through ion–ion reactions, PTCR concentrates the signal that was previously split across multiple charge channels, allowing these low-abundance fragments to be confidently identified. Fragments found in the runs without PTCR are usually also identified with PTCR (Figure S5, Supporting Information). However, additional fragments from the PTCR runs have been found to have higher masses, providing information on larger pieces of the sequence. For the light chain, N-terminal fragments identified with PTCR only are mainly located between amino acids 195 and 213 (C-terminal). In contrast, for the heavy chain, most of them were located close to the hinge region, between amino acids 200–220 (N-term fragments) and 250–270 (C-term fragments), as shown in Figure 5A. By looking deeper into the fragment ions identified, we can also notice the differences in the charge distribution between those identified without and with PTCR (Figure 5B). For both chains, fragment ions identified with and without PTCR are mostly charge states 4+ to 6+, while those identified only without PTCR are predominantly between charge states 5+ to 7+, and those identified with PTCR only between charge states 2+ to 5+. When examining the fragment ions from PTCR alone, a cluster of high charge-state ions (5+ to 7+) was also observed for both chains. Those higher charge state ions from PTCR lead to the identification of higher mass fragments, which provide information about the middle of the heavy chain sequence and the light chain C-terminus. Indeed, large fragments mapping the middle of the sequence have high mass and high charge state, resulting in signal dilution across multiple channels in the isotopic profile, and potentially preventing the fragment ion from passing the S/N filter. When applied, PTCR lowers the fragment ion charge that would otherwise be distributed across multiple channels. PTCR increases the S/N of fragments already present in the spectrum, enabling them to pass the S/N threshold for deconvolution and then allowing their confident assignment.^69^

**Figure 5:**
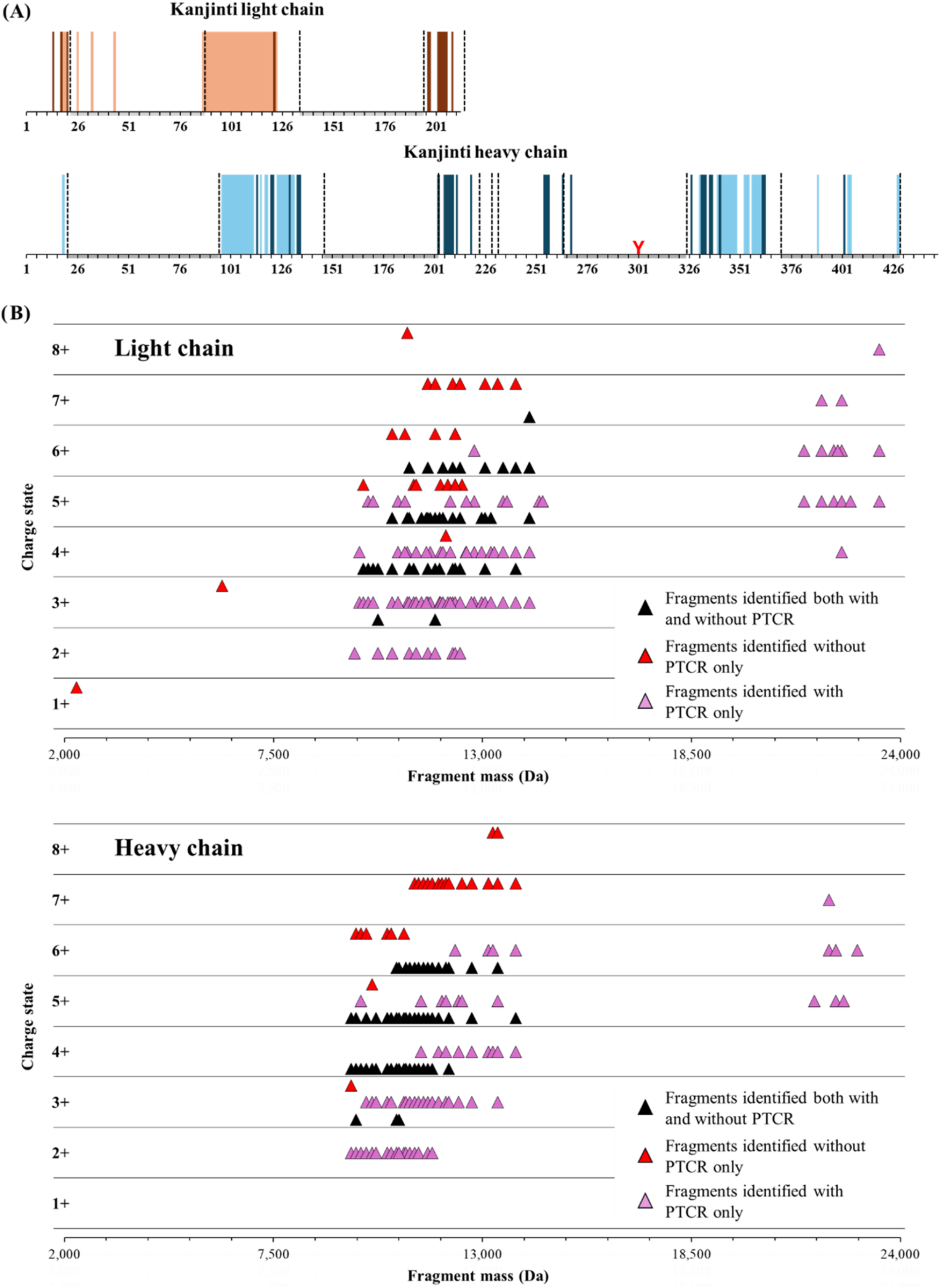
(A) Bar code fragmentation maps of trastuzumab biosimilar Kanjinti light and heavy chains obtained after ETD and UVPD fragmentation. Light color bars represent the fragment identified without PTCR while the ones in dark color represent fragment ion identified only thanks to PTCR runs (i.e., UVPD 30 ms + PTCR 20 ms and ETD 25 ms + PTCR 20 ms). (B) Distribution of the mass and the charge of the identified fragments in Herceptin heavy and light chains using ETD 25 ms with and without PTCR. Fragments identified in both runs are displayed in black, those only identified without PTCR are in red, and those only identified with PTCR are in purple. To be considered identical, fragments must have the same mass and the same charge state.

### 3.4 Identification of PTMs on Intact Trastuzumab by Native TD-MS

The characterization of mAbs usually includes the identification of PTMs, as well as their location within the sequence. Indeed, the same modification could have different impacts on the safety or immunogenicity of the molecule. For example, deamidation in CDR regions has a significantly greater impact on the potency of the mAb compared to other locations in the sequence.^87,88^ Here, we utilized charge variant separation to separate the different proteoforms, which were then submitted to native TD. Among the proteoforms identified, deamidated trastuzumab is the most abundant (around 8%, Figure 1B). Deamidation is only a +1 Da modification; the *m*/*z* distinction between a deamidated and nondeamidated fragment is minimal, and only a shift of the apex of the isotopic profile allows differentiation between them, which is not possible when the two species are analyzed at the same time. However, here we performed native TD during elution of the deamidated peak (8.5–10 min) to examine the characteristic fragment ions of the deamidated species and compared them with those of the main peak. One of the most well-known and critical hotspots for deamidation in trastuzumab is Asn 30 in the light chain, and deamidation at this location was confirmed by peptide mapping (data not shown). Then, fragment ions generated during each elution peak were searched (deamidated and nondeamidated) against the corresponding sequence. Figure 6 shows an example of the same fragment ions from ETD and HCD fragmentations, generated either during the elution of the deamidated trastuzumab or the main peak. As described, for both fragmentation techniques, the theoretical profile of the expected fragment ions (triangles) matches perfectly with the experimental data (circles) for each isotopic profile. As expected, a slight shift in the apex of the isotopic profile is observed for the fragment ions of deamidated trastuzumab due to the +1 Da modification. For both species (i.e., deamidated and nondeamidated), similar S/N ratios and scores are also obtained. This example demonstrates the ability of our approach to discriminate and identify.

**Figure 6:**
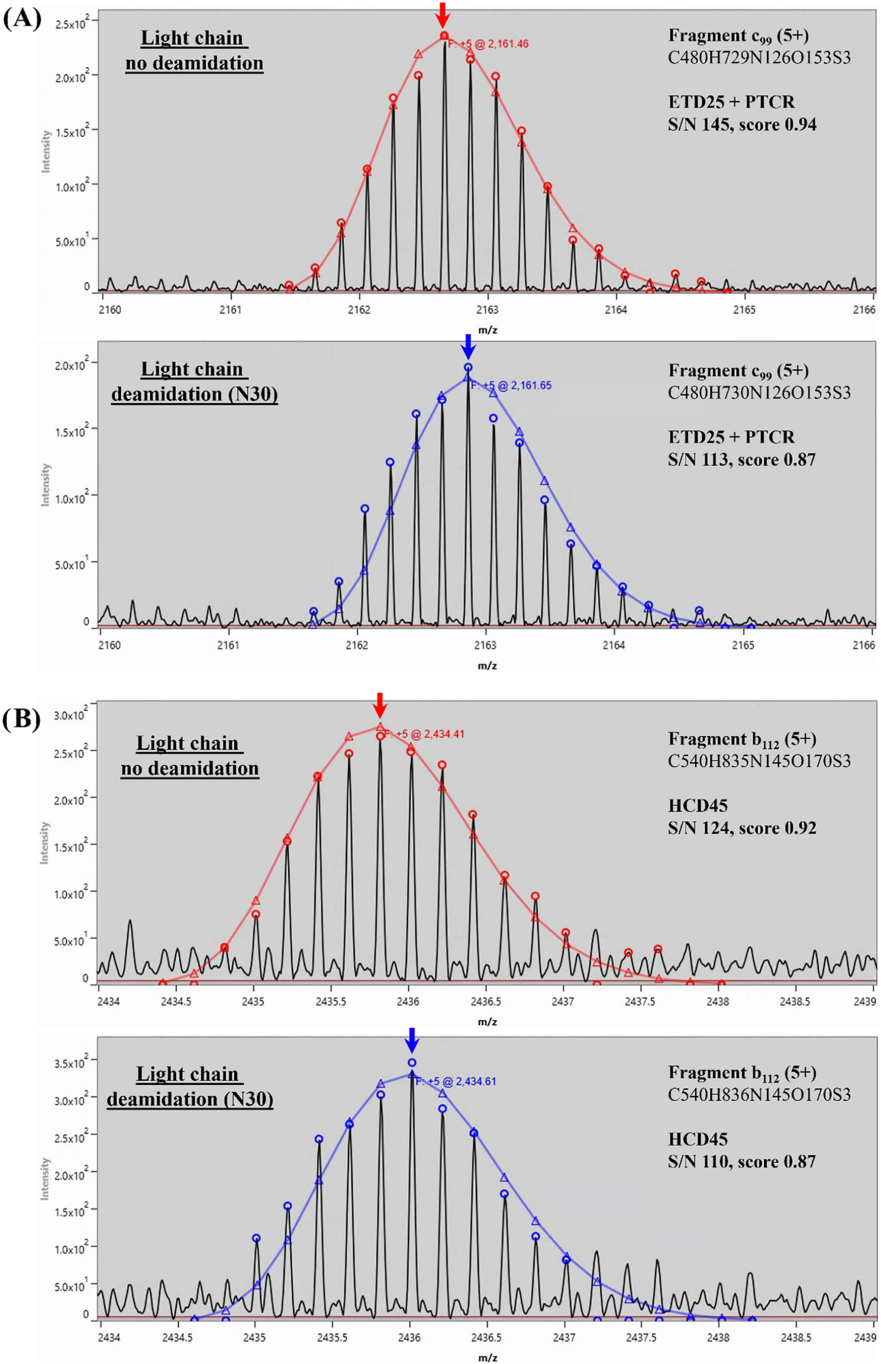
Examples of Herceptin fragment ions identified in different chromatographic peaks. Red isotopic profiles correspond to fragments ions from the main peak (no deamidation, 12–13.5 min) and blue to fragment ions from the first chromatographic peak (trastuzumab + 1 × deamidation, 8.5–10 min). (A) Isotopic profile of fragment c_99_ (5+) from ETD 25 ms + PTCR run. (B) Isotopic profile of fragment b_112_ (5+) from HCD 45 run. Triangles represent the theoretical isotopic profile of the identified fragments. Arrows show the apex of the isotopic profile and *m*/*z* values in blue and red represent the monoisotopic mass of the identified fragment.

Like deamidation, other PTMs can also be identified by native TD. For example, pyro-Glu modification at the N-terminus of the heavy chain (−17 Da) or succinimide intermediate at Asn 55 (−18 Da) of the heavy chain has been investigated using our native TD approach. For both PTMs, multiple fragment ions were identified, confirming the presence of those modifications at their expected location on the heavy chain. For example, fragment ions b_102_ (6+) and b_115_ (7+) have been identified after HCD fragmentation over the elution peak of the trastuzumab with pyro-Glu. Those fragments confirm the presence of the pyro-Glu modification on the N-terminal of the heavy chain. Likewise, fragment ions characteristic of the succinimide modification on Asn 55 of the heavy chain have been found. A few of those ions, from both HCD and ETD fragmentations, are shown in Figures S6 and S7 (Supporting Information). Those examples illustrate the ability of the native TD approach to identify fragment ions, confirming the presence of succinimide at Asn 55 of the heavy chain.

With all these examples, we demonstrate that the hyphenation of charge variant analysis to the native TD-MS approach is a robust workflow that can provide information on the PTMs present on the intact molecule and offer insights into their locations. As a proof of concept, we identified deamidation on trastuzumab light chain, as well as pyro-Glu and succinimide on the heavy chain. However, this approach still has some limitations. Under native conditions, when the intact molecule is fragmented and still contains disulfide bridges, extensive sequencing is not possible, thereby preventing the amino acid-level location of the PTMs. Indeed, that would require being able to fragment the sequence between each amino acid. Nevertheless, this approach remains helpful for gaining insights into the region of the sequence where PTMs are located, without the need for time-consuming peptide mapping experiments. In addition, the fragmentation of low-abundance proteoforms may lead to very low-intensity fragments that may not meet the S/N ratio threshold for identification by the software. However, it opens the door to further applications, especially for bispecific and multispecific antibodies, where the location of PTMs on one or the other part of the molecule may be critical.

## 4 Conclusions

The present work describes the successful coupling of pH-gradient charge variant analysis with native TD-MS for the characterization of trastuzumab. This workflow enables chromatographic separation of individual charge variants under native conditions and the comparison of proteoforms abundance between trastuzumab originator and biosimilars. Then, using proteoform-specific fragmentation on the LC–MS time scale by combining complementary dissociation methods (HCD, ETD, and UVPD), we achieved reproducible sequence coverage across trastuzumab samples for both heavy and light chains, as well as complete sequencing of both CDR3 regions. The use of PTCR led to the identification of high-mass fragments that were not detected without PTCR, thereby improving sequence coverage and providing information on the middle of the heavy chain sequence and the C-terminus of the light chain. Importantly, this approach also provides insights about the location of key PTMs, such as deamidation, succinimide, and N-terminal pyroGlu, directly on intact proteoforms from charge variant analysis, without requiring peptide-level digestion.

Although limitations regarding the use of the native TD workflow remain, such as restricted fragmentation of the sequence within the disulfide bridges and a limited number of charge states available for fragmentation, our results demonstrate the potential of this approach to provide rapid and informative insights about intact mAb in their native state and is complementary to middle-down MS and peptide mapping. Indeed, peptide mapping helps provide information about the location of individual PTMs, but information about potentially coexisting PTMs at the intact level is lost during the digestion into peptides. Here, by fragmenting the intact molecule, co-occurring PTMs on a single proteoform can be identified simultaneously. While this study is a proof-of-concept for a standard mAb, it opens the way for characterizing increasingly complex antibody formats, such as bispecifics or multispecifics, for identifying chain-specific PTMs, for example. Overall, this work establishes native TD-MS coupled with charge variant separation as a robust and highly informative platform for detailed charge heterogeneity analysis in therapeutic antibodies, with broad applicability across biopharmaceutical characterization.

## Supplementary Material





## Data Availability

Data of this study is available from the corresponding author upon reasonable request. The raw mass spectrometry data has been deposited to ProteomeXchange Consortium via the MassIVE partner repository under the accession: MSV000100790.

## Notes

The following files are available free of charge. The Supporting Information is available free of charge at https://pubs.acs.org/doi/10.1021/jasms.5c00438.

Tables reporting data processing parameters and intact mass results; seven supporting figures show peptides mapping results for N30 deamidation, N55 succinimide, and glycation, detailed sequence coverage for each sample, examples of raw fragmentation spectra, Venn diagram comparing identified fragment ions with and without PTCR, and isotopic profiles of fragment ions confirming pyroGlu and succinimide modifications (PDF)
MS deconvolution results (Table S3) (XLSX)
