# Supplementary material for: Native Top-Down Mass Spectrometry Combined with High-Resolution Charge Variant Analysis of Trastuzumab Originator and Biosimilars
Source: J Am Soc Mass Spectrom. 2026 Apr 17;37(5):1173–86. doi: 10.1021/jasms.5c00438 (PMC13154209; doi:10.1021/jasms.5c00438)

Native Top-Down Mass Spectrometry combined with High-Resolution Charge Variant Analysis of Trastuzumab Originator and Biosimilars

*Corentin Beaumal [#], Kristina Srzentić [■], Sara Carillo [#], Jonathan Bones [#, ♦, *].*

Characterization and Comparability Laboratory, NIBRT – National Institute for Bioprocessing Research and Training, Foster Avenue, Mount Merrion, Blackrock, Dublin, A94 X099, Ireland

■ Thermo Fisher Scientific, 11 Neuhofstrasse, 4153 Reinach, Switzerland

♦ School of Chemical and Bioprocess Engineering, University College Dublin, Belfield, Dublin D04 V1W8, Ireland

* Corresponding author: Jonathan Bones, jonathan.bones@nibrt.ie.

Supplementary data

Supplementary Table S1: Sliding Window Deconvolution parameters for analysis of CVA intact protein data in the BioPharma Finder 3.1 software.

**Chromatogram parameters**	**Setting**
m/z Range	4,500-7,000
Chromatogram Trace Type	TIC
**Source Spectra method**	**Setting**
Sliding Window	
RT range	7.0-17.0 min
Target Avg Spectrum Width	0.2 min
Target Avg Spectrum Scan Offset	2
Merged Tolerance	10 ppm
Max RT Gap	0.2 min
Min. Number of Detected Intervals	3
**Deconvolution Algorithm**	**Setting**
ReSpect™ (Isotopically Unresolved)	
Model Mass Range	145,000-150,000
Deconvolution Mass Tolerance	10 ppm
Charge State Range	10 to 35
Minimum Adjacent Charges (low & high model mass)	4 - 4

Supplementary Table S2: Elution time windows used for the characterisation of each proteoform.

**Species**	**Start time (min)**	**Stop time (min)**
Trastuzumab, 1 × deamidation	8.5	10.0
Trastuzumab	12	13.5
Trastuzumab, 1 × pyroGlu	13.5	14.6
Trastuzumab, 1 × succinimide	14.6	15.8

Supplementary Table S3: MS deconvolution results.

See Excel file.

Supplementary Figure S1: Relative intensity of N30 light chain deamidation and N55 heavy chain succinimide from peptide mapping. Quantitative analysis was obtained from triplicate technical replicates.

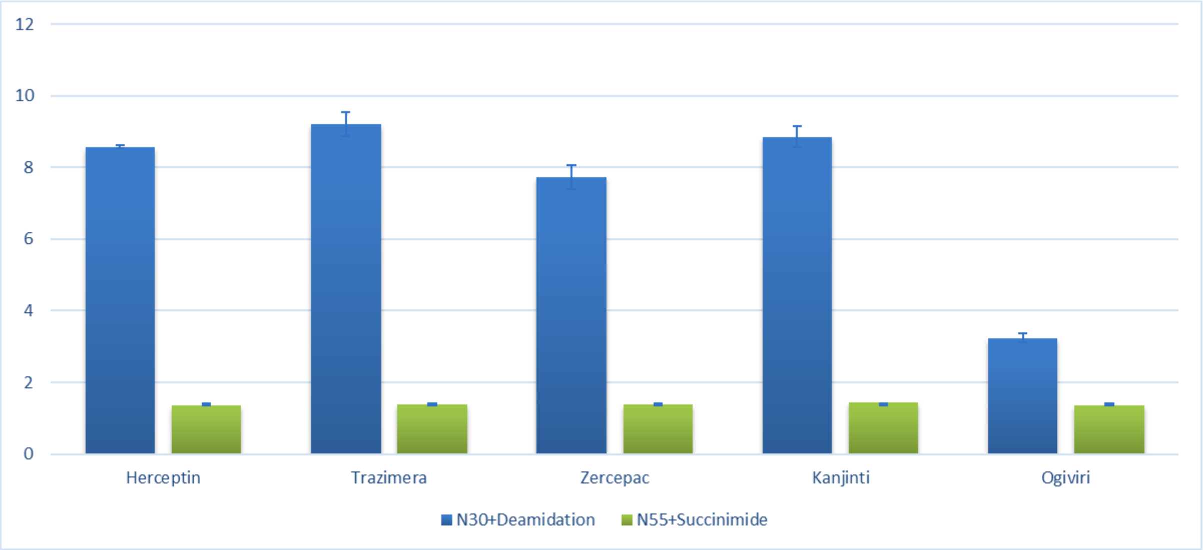

Supplementary Figure S2: a) relative abundance of lysine glycation obtained through peptide mapping analysis. b) relative abundance of N300 N-glycans obtained through peptide mapping analysis. Quantitative analysis was obtained from triplicate technical replicates. Only species present in at least one of the samples with an abundance above 0.5% are displayed.

a)

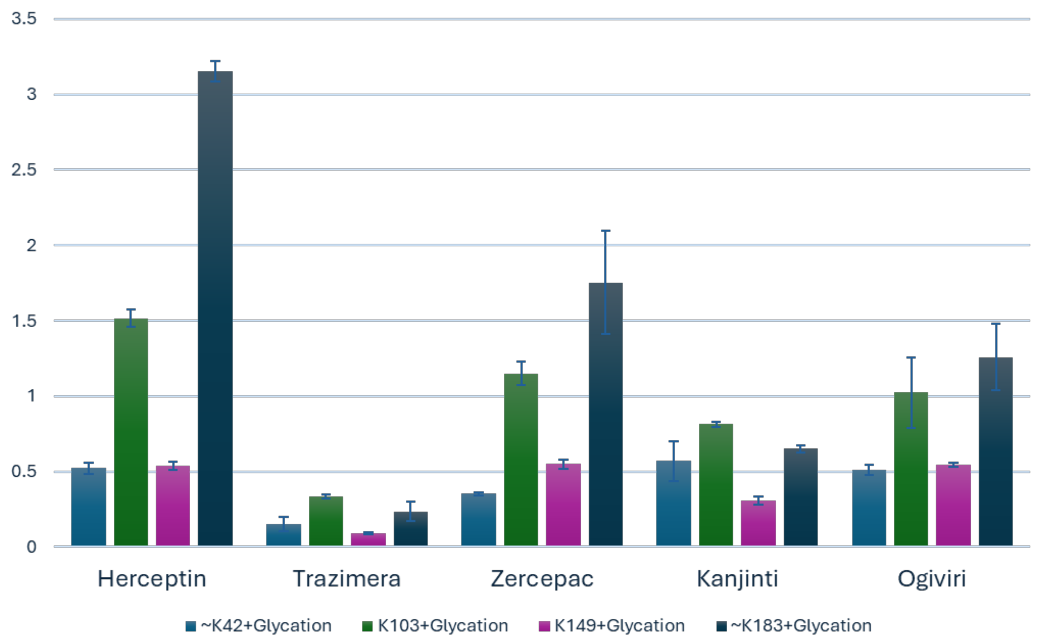

b)

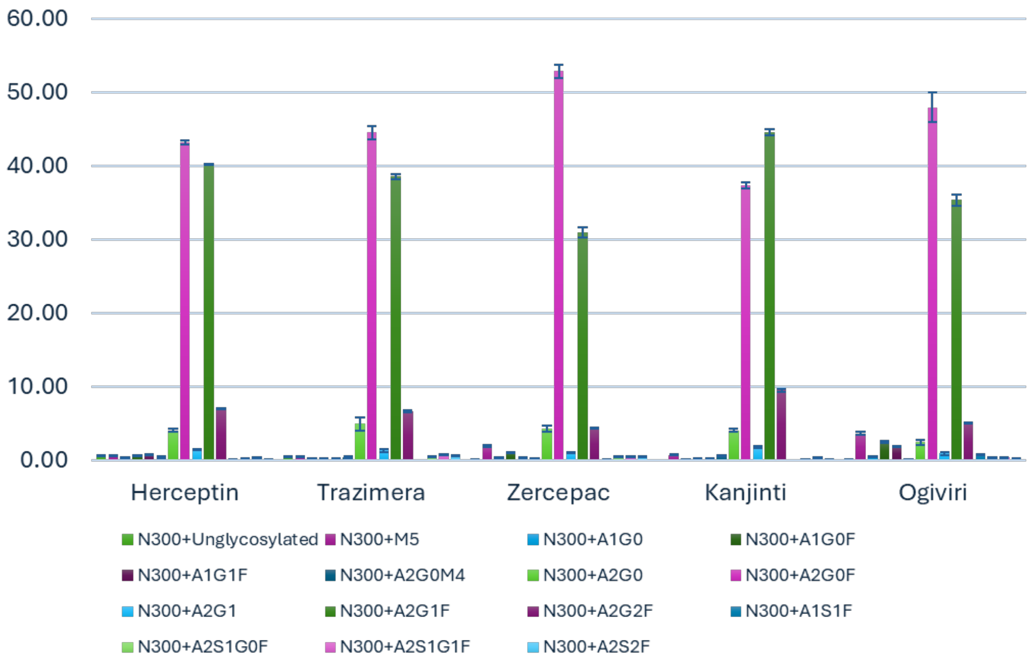

Supplementary Figure S3: Examples of raw fragmentation spectra obtain from Herceptin fragmentation over the main chromatographic peak using (A) HCD 45% NCE, (B) UVPD 30 ms, (C) ETD 25 ms, and (D) ETD 25 ms + PTCR 20 ms.

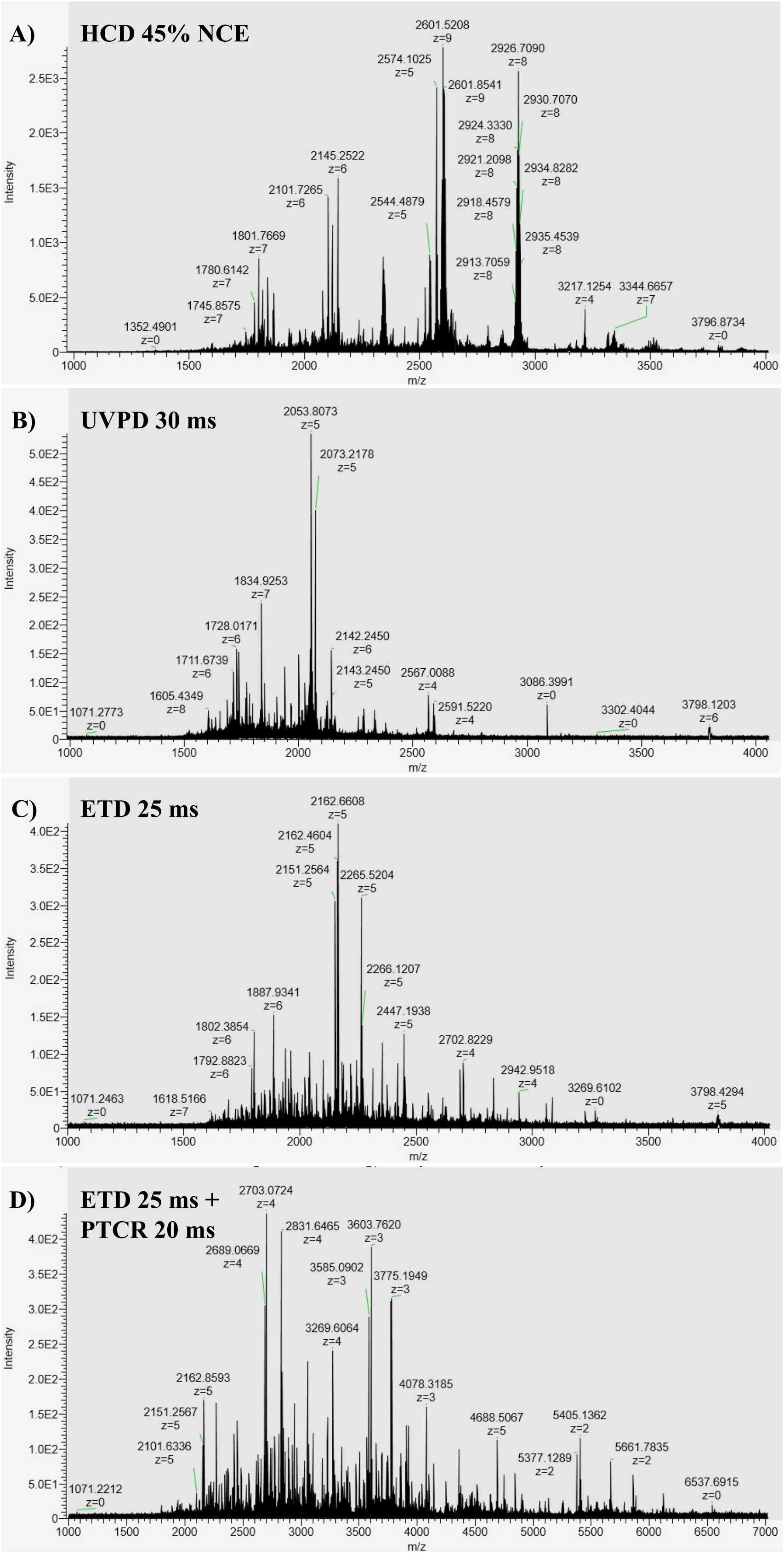

Supplementary Figure S4:

Bar plots representing the sequence coverage obtained for the light and heavy chains of each sample, by each fragmentation used in this study as well as the sequence coverage after combination of all the fragmentation together (n=8).

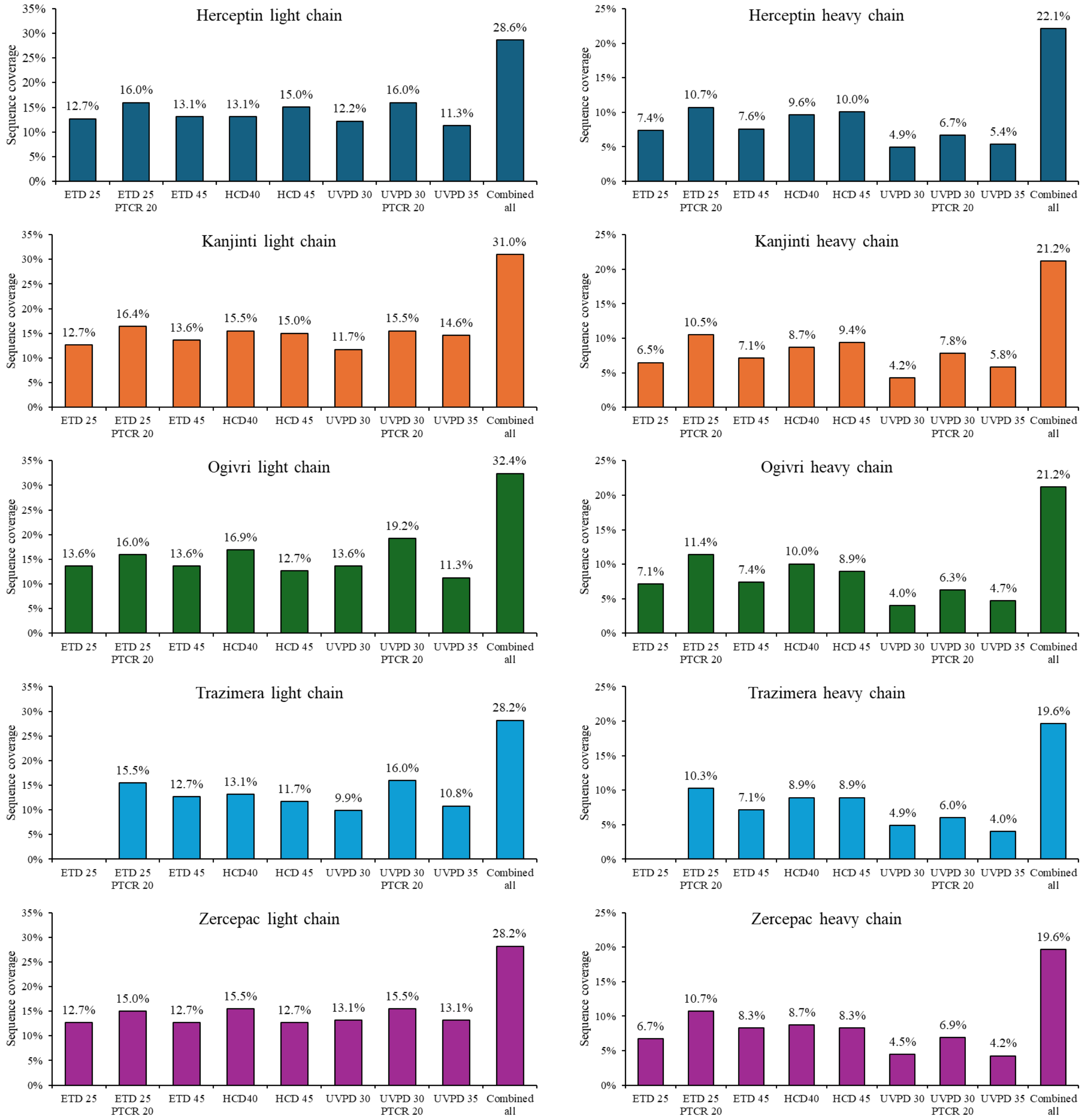

Supplementary Figure S5: Venn diagram representing the fragments identified with and without PTCR after ETD and UVPD for Herceptin heavy and light chains.

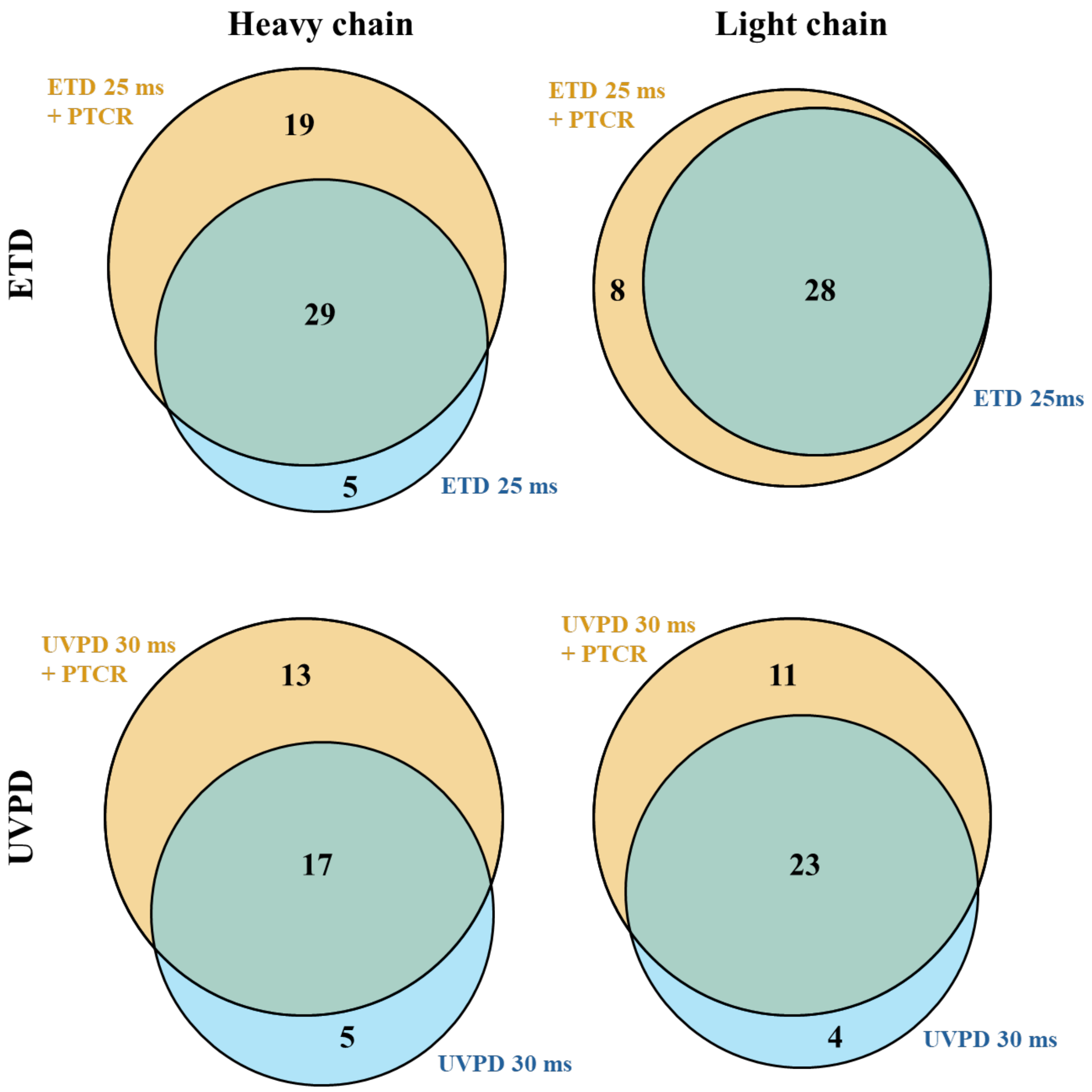

Supplementary Figure S6: Examples of isotopic patterns of fragment ions identified in the Herceptin elution peak of trastuzumab + 1 × succinimide. Right panels represent for the same fragment ion (i.e., same cleavage site and charge state) both isotopic patterns of the trastuzumab heavy chain with the succinimide modification (light green) and the trastuzumab heavy chain without modification (dark green). Left panels represent a zoom on the isotopic pattern of the trastuzumab heavy chain with succinimide modification and m/z values in light green and dark green represent the monoisotopic mass of the identified fragments.

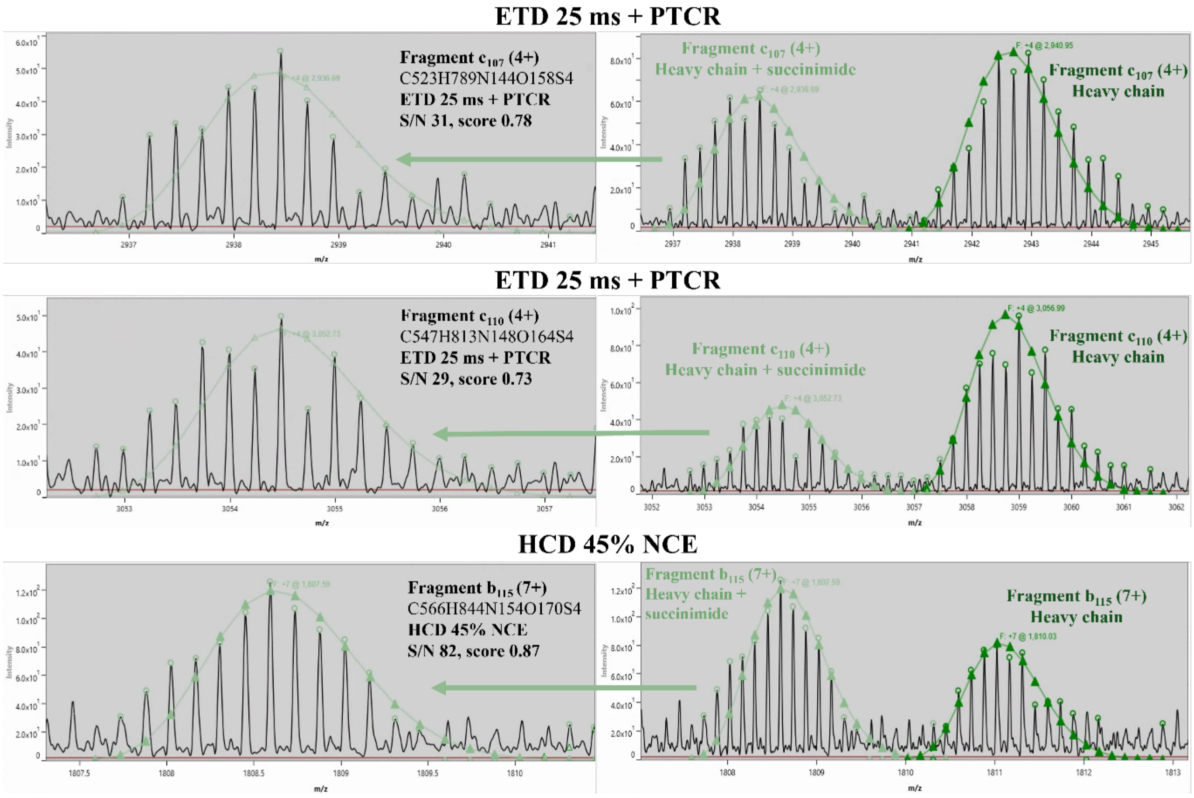

Supplementary Figure S7: Examples of isotopic patterns of fragment ions identified in the Herceptin elution peak of trastuzumab + 1 × pyroGlu. Right panels represent for the same fragment ion (i.e., same cleavage site and charge state) both isotopic patterns of the trastuzumab heavy chain with the pyroGlu modification (orange) and the trastuzumab heavy chain without modification (dark green). Left panels represent a zoom on the isotopic pattern of the trastuzumab heavy chain with pyroGlu modification and m/z values in orange and dark green represent the monoisotopic mass of the identified fragments.

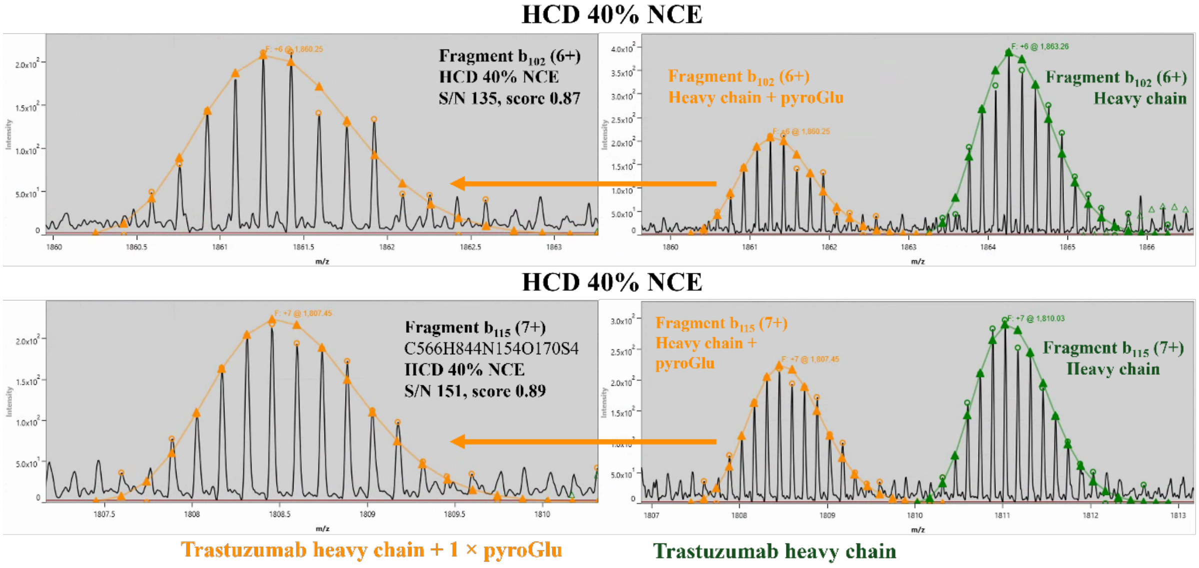

Supplement: Supplementary file 1 [file js5c00438_si_001.pdf]
